# Interfacial Tension Measurements in Microfluidic Quasi-Static Extensional Flows

**DOI:** 10.3390/mi12030272

**Published:** 2021-03-06

**Authors:** Doojin Lee, Amy Q. Shen

**Affiliations:** 1 Department of Polymer Science and Engineering, Chonnam National University, Gwangju 61186, Korea; 2 Micro/Bio/Nanofluidics Unit, Okinawa Institute of Science and Technology Graduate University, Onna, Okinawa 904-0495, Japan

**Keywords:** interfacial tension, quasi-static extensional flows, droplet microfluidics, droplet deformation

## Abstract

Droplet microfluidics provides a versatile tool for measuring interfacial tensions between two immiscible fluids owing to its abilities of fast response, enhanced throughput, portability and easy manipulations of fluid compositions, comparing to conventional techniques. Purely homogeneous extension in the microfluidic device is desirable to measure the interfacial tension because the flow field enables symmetric droplet deformation along the outflow direction. To do so, we designed a microfluidic device consisting of a droplet production region to first generate emulsion droplets at a flow-focusing area. The droplets are then trapped at a stagnation point in the cross junction area, subsequently being stretched along the outflow direction under the extensional flow. These droplets in the device are either confined or unconfined in the channel walls depending on the channel height, which yields different droplet deformations. To calculate the interfacial tension for confined and unconfined droplet cases, quasi-static 2D Darcy approximation model and quasi-static 3D small deformation model are used. For the confined droplet case under the extensional flow, an effective viscosity of the two immiscible fluids, accounting for the viscosity ratio of continuous and dispersed phases, captures the droplet deformation well. However, the 2D model is limited to the case where the droplet is confined in the channel walls and deforms two-dimensionally. For the unconfined droplet case, the 3D model provides more robust estimates than the 2D model. We demonstrate that both 2D and 3D models provide good interfacial tension measurements under quasi-static extensional flows in comparison with the conventional pendant drop method.

## 1. Introduction

Interfacial tension (IFT) between immiscible liquids plays an important role in determining the morphology, stability, structure and dynamics of emulsions and multiphase systems that are used in various scientific and industrial applications, such as oil recovery, cosmetics, pharmaceuticals and food processing [1,2,3,4,5]. Droplet microfluidics technology is becoming increasingly important for high throughput processes such as polymerase chain reaction (PCR), gene sequencing, molecular detection, drug delivery and disease diagnostics [6,7]. Droplet microfluidics enables handling and analysis of a wide range of samples via the generation and manipulation of discrete droplets in micro-devices [8,9,10]. Droplet microfluidic systems can provide compartmentalized droplet reactors for enhanced mixing and mass transfer within reduced diffusion time and distance. This method involves highly monodispersed droplets on the order of sub-micrometer to hundreds of micrometers in diameter, with high production rates up to thousands of droplets per second. Various functions have been realized in droplet microfluidic systems, including identification and quantification, screening and real-time monitoring of various biological entities. In addition, droplet microfluidics can also generate non-spherical particles, double emulsions, hollow microcapsules and micro-bubbles which are useful in a wide range of applications, including the synthesis of biomolecules, drug delivery, single-cell analysis, food and feed industry and diagnostic testing [11,12,13]. Recent advancements of measuring IFT using droplet microfluidics has offered advantages of small sample volume and reagent consumptions, portability, minimum contamination and easy manipulations of fluid compositions [14,15,16,17,18]. More importantly, the small length scales of microfluidic flows enable fast response and enhanced throughput for IFT measurements, which offers a new venue for the development of “lab-on-a-chip-tensiometer” [14,19]. IFTs in microfluidic platforms can be calculated by either balancing interfacial tension and drag force acting on droplets [16,20,21,22], or detecting pressure drop and contact angle between two immiscible liquid streams in a tapered microchannel [14,18,23]. Of these two approaches, the force balance based droplet deformation method normally gives more accurate and reproducible results than the pressure drop method.

For microfluidic approaches relying on the dynamic droplet deformation to measure IFTs, both empirical scaling analysis [22] and theoretical models [16,20] have been reported in literature. The empirical scaling shows that IFT follows a power law relationship with respect to the droplet deformation, the droplet size, the flow velocity and the continuous phase viscosity [22], which provides a reasonable estimate for IFTs. Tuning the separation distance of confined droplets and merging of confined droplets were achieved by varying the IFTs in a uniform cross-section capillary [7]. Another microfluidic approach showed that the IFTs could be extracted by the deformation of individual emulsion droplets under steady state shear flow at high surfactant concentrations [4]. However, knowledge on the relationship between the droplet confinement and IFT is still lacking. Hudson et al. developed a novel dynamic droplet microfluidic platform to measure IFTs by employing the Rallison’s droplet deformation theory [24] in which a spherical droplet deforms to an elongated ellipsoidal shape due to the extensional stresses acting on the droplet [16,20]. This method has advantages of enabling quantitative real-time analysis of droplet deformation to calculate IFTs. It assumes that purely extensional flow takes place in a constriction region in a microchannel to generate homogeneous extensional dynamic droplet deformation. However, such condition is difficult to achieve in straight microchannels since abrupt constrictions of a channel create a combination of shear close to the walls and an inhomogeneous extensional flow along the centerline region [25].

Homogeneous extension for dynamic droplet deformation can be approximated with an optimized microfluidic constriction geometry, however, it requires robust optimization procedures through computational fluid dynamic (CFD) simulation [26]. An alternative approach is to use the cross junction (also referred to as the cross-slot flow) flow geometry [27,28,29,30], which has the advantage of producing a better approximation to 2D extensional flow. Furthermore, the cross junction flow field contains a stagnation point where residence times are elevated and steady state deformation can be achieved. Understanding the influence of confinement to the droplet deformation under homogeneous extensional flows will extend the utility of this approach to reveal droplet dynamics such as surfactant adsorption, droplet aging and transient interfacial dynamics. Recently, Narayan et al. [31] studied the droplet shape relaxation in a four-channel microfluidic hydrodynamic trap in which they trapped and controlled the position of droplets using hydrodynamic forces and investigated the droplet shape relaxation after cessation of the pressure pulse. An empirical scaling relationship between the droplet shape relaxation and droplet radius was subsequently established and compared to the characteristic relaxation time for a droplet relaxing to equilibrium in a quiescent and infinite fluid reservoir.

For confined droplets in a shallow microchannel, the fluid flow with a confined droplet can be described by the Darcy approximation in which the droplet deformation is associated with the Capillary number $Ca=\eta_{c}\dot{\varepsilon }R_{0}/\sigma$ and the channel confinement parameter $\delta =2R_{0}/h$, where $\eta_{c}$, $\dot{\varepsilon }$, σ, $R_{0}$ and *h* are the continuous phase viscosity, the strain rate, the interfacial tension, the initial droplet radius and the channel height, respectively [32]. All the mathematical symbols used in this study and their definitions are listed in Table 1. The confined droplet case is useful in certain applications where the channel geometry is fixed and the droplet sizes are comparably larger than the channel height. For unconfined droplets, the droplet deformation under shear flow and extensional flow is well understood (more details in Section 2) [24,33,34]. We assume that the droplet is subjected to quasi-static extensional flows at the cross junction wherein the velocity of the fluid around the droplet is instantaneously and uniquely determined by the droplet shape and the imposed flow. The cross junction channel is used and the effect of confinement is explored by varying the droplet size relative to the channel height. We combine experiments and modeling to validate the measurement of IFTs in microfluidic quasi-static 2D and 3D flows. We use the quasi-static 2D Darcy approximation (confined droplet case) and 3D small deformation (unconfined droplet case) models to estimate IFTs and compare the IFT results measured by the conventional pendant drop method. Our microfluidic droplet and cross junction platform not only provides emulsion production capacities, but also incorporates real-time, in-situ interfacial tension measurement on a single chip.

## 2. Theoretical Background

### 2.1. 2D Darcy Approximation Model for a Confined Droplet

**Figure 1:**
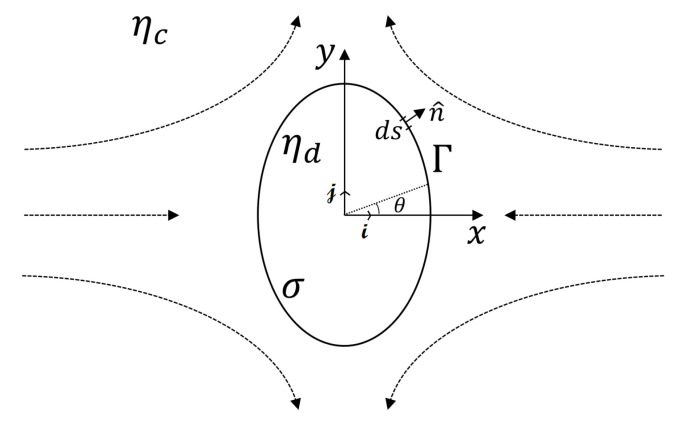
Schematic illustration of a droplet with initial radius $R_{0}$ deformed to an elliptical shape when subjected to a quasi-static extensional flow.

For the 2D confined droplet deformation model, we consider the droplet deformation in the presence of quasi-static extensional flows at the cross junction, wherein the depth-averaged velocity of the fluid $\vec{u}$ around the droplet is instantaneously and uniquely determined by the droplet shape and the imposed flow (Figure 1). The droplet is surrounded by the continuous phase, with the interface Γ defining its shape. The viscosities of the dispersed and continuous phases, and the interfacial tension are denoted by $\eta_{d}$, $\eta_{c}$ and σ, respectively. When the droplets are confined in the microfluidic channel under the flow, the geometrical constraint of the channel walls can affect the droplet deformation and relaxation [32,35]. The degree of confinement is defined by the channel confinement parameter $\delta =2R_{0}/h$, with $R_{0}$ the initial droplet radius and *h* the channel height.

Taylor’s pioneering work described the deformation of unconfined droplets in shear flows, with the droplet deformation being described by $D∼Ca\frac{19\eta_{d}+16\eta_{c}}{16\eta_{d}+16\eta_{c}}$, where D=(l−b)/(l+b) is the droplet deformation parameter, *l* and *b* are the largest and smallest distances of the droplet surface from its center, $Ca=\eta_{c}\dot{\varepsilon }R_{0}/\sigma$ is the capillary number, $\dot{\varepsilon }$ is the strain rate, $\eta_{c}$ and $\eta_{d}$ are the continuous and dispersed phases, respectively. When the confinement effect is taken into account for a confined droplet, the droplet deformation should also depend on the confinement parameter δ. The effect of confinement on the droplet deformation has been studied theoretically in cases with δ<1, in which Darcy approximation of the Stokes flow in a shallow geometry microchannel was described as [32]

(1) $$\vec{u}=-\left(h^{2}/12\eta_{c}\right)\nabla p,$$

where $\vec{u}=u\left(x,y\right)\hat{i}+v\left(x,y\right)\hat{j}$ is the depth-averaged velocity field, and *p* is the pressure. For an incompressible flow, the Stokes equation can be solved by using a complex potential w(z)=ϕ(x,y)+iψ(x,y), where z=x+iy is the complex variable, $\phi \left(x,y\right)=-h^{2}p\left(x,y\right)/12\eta_{c}$ is the velocity potential and ψ(x,y) is the 2D stream function. The gradient of the velocity potential $w^{\prime }\left(z\right)$ is related to the fluid velocity. Assuming that the velocity is continuous across the droplet interface, $w_{c}^{\prime }\left(z\right)=w_{d}^{\prime }\left(z\right)$ for *z* on the interface Γ. The problem is simplified by assuming $\eta_{c}=\eta_{d}=\eta$. With this, the velocity potential for any z can be computed by the Sokhotski–Plemelj formula as [32,36]

(2) $$w\left(z\right)=\frac{h^{2}\sigma }{24\pi \eta }\int_{\Gamma }\frac{dt/ds}{{\left(t-z\right)}^{2}}dt-\frac{\dot{\varepsilon }z^{2}}{2},$$

where dt and ds are the infinitesimal lengths of the droplet thickness and surface. There are two contributions to the net force acting on the droplet. The interfacial tension σ preserves the spherical droplet shape from the deformation whereas the viscous drag force upon extensional flows elongates the droplet. The net interfacial tension force is obtained by integrating interfacial tensions acting on the interface tangentially (first term of the right hand side (RHS) in Equation (2)). It is assumed that the extensional strain rate $\dot{\varepsilon }$ is proportional to the net viscous drag force acting on the droplet along the normal direction, which counteracts against the net interfacial tension force (second term of RHS in Equation (2)).

Assuming that the droplet deforms to the elliptical shape, the droplet deformation is evaluated by the integration of the stress balance on the interface as [32,36]

(3) $$\frac{-1}{3\pi A}\int_{\Gamma }\frac{dt}{ds}dt=\frac{Ca\delta^{2}}{\sqrt{1-D^{2}}},$$

where, *A* and $\dot{\varepsilon }$ are the area of the droplet and the strain rate. This equation involves the capillary number Ca and the confinement parameter δ. The left hand side (LHS) in Equation (3) can be expressed by the integral of the droplet deformation with respect to an angle at the surface of the ellipse [32].

(4) $$\frac{1}{\delta^{2}}\frac{\sqrt{1-D^{2}}}{3\pi }\int_{0}^{2\pi }\frac{2D+(1+D^{2})\cos2\theta }{\sqrt{1+D^{2}+2D\cos2\theta }}d\theta =Ca,$$

Equation (4) can be solved numerically to obtain Ca for any measurable *D* and confinement ratio δ. *D* can be measured experimentally by using optical microscopy, and Ca is determined based on Equation (4). The interfacial tension σ is then extracted from $Ca=\eta \dot{\varepsilon }R_{0}/\sigma$. In fact, the capillary number Ca in Equation (4) contains a mean viscosity based on the assumption that $\eta_{d}$ = $\eta_{c}$ = η to avoid mathematical difficulty. However, this oversimplification may cause inaccurate determination of Ca. Therefore, we introduce an effective viscosity $\eta_{e}$ to calculate the capillary number of Equation (4) and compare the results with those obtained by using the mean viscosity. Here, the effective viscosity is expressed as [33]

(5) $$\eta_{e}=\alpha \eta_{c}=\frac{\left(2\hat{\eta }+3\right)\left(19\hat{\eta }+16\right)}{40(\hat{\eta }+1)}\eta_{c},$$

where $\hat{\eta }=\eta_{d}/\eta_{c}$ is the relative viscosity between the dispersed ($\eta_{d}$) and continuous ($\eta_{c}$) phases. Since all the parameters *D*, $\eta_{e}$, $\eta_{c}$, $\dot{\varepsilon }$, $R_{0}$ can be measured experimentally and Ca is obtained by using Equation (4), we can subsequently estimate the interfacial tension with known Ca. The interfacial tensions are calculated by using either the effective viscosity or the mean viscosity as $\sigma_{1}=\eta_{e}\dot{\varepsilon }R_{0}/Ca$ or $\sigma_{2}=\eta_{c}\dot{\varepsilon }R_{0}/Ca$. We will compare the interfacial tension results obtained by using $\eta_{e}$ or $\eta_{c}$ with the pendant drop method in Section 4.

### 2.2. 3D Small Deformation Model for an Unconfined Droplet

An early study for the droplet deformation under extensional flows was established by Taylor where a four-roller apparatus was used to achieve a distortion of a droplet by viscous forces associated with a continuous phase fluid [33]. The deformation and dynamics of a small viscous droplet surrounded by a continuous phase fluid under extensional flows was further studied by Rallison [24] and Hudson [16,20], which is expressed as

(6) $$\frac{\partial D}{\partial t}=\frac{5}{2\hat{\eta }+3}\dot{\varepsilon }-\sigma \frac{D}{\eta_{e}R_{0}},$$

where $R_{0}$ is the initial droplet radius, *D* is the droplet deformation, *t* is the time, $\hat{\eta }=\eta_{d}/\eta_{c}$ is the relative viscosity, $\eta_{e}$ is the effective viscosity and $\dot{\varepsilon }$ is the strain rate, respectively.

The instantaneous deformation of a droplet can be expressed in a convenient form by considering time-invariant extensional flows with the extensional strain rate $\dot{\varepsilon }=du/dx$ and $\frac{\partial D}{dt}=\frac{\partial D}{\partial x}\frac{\partial x}{\partial t}=u\frac{\partial D}{\partial x}$, which is expressed as

(7) $$\;\eta_{e}\left(\frac{5}{2\hat{\eta }+3}\dot{\varepsilon }-u\frac{\partial D}{\partial x}\right)=\sigma \left(\frac{D}{R_{0}}\right),$$

The first term on the left hand side (LHS) in Equation (7) accounts for the steady-state deformation and is primarily governed by $\dot{\varepsilon }$. The second term corresponds to the time dependent droplet deformation. With our microfluidic platform, the second term $u\frac{\partial D}{\partial x}$ vanishes since the droplet is trapped at the center of the cross junction and deforms quasi-statically. Based on simplified $\eta_{e}\left(\frac{5}{2\hat{\eta }+3}\dot{\varepsilon }\right)=\sigma \left(\frac{D}{R_{0}}\right)$, experimentally we can plot $\eta_{e}\left(\frac{5}{2\hat{\eta }+3}\dot{\varepsilon }\right)$ against $\frac{D}{R_{0}}$ to extract the slope σ while varying $\dot{\varepsilon }$. For Newtonian fluids, this relationship is valid as long as *D* < 0.15 for modest deformations before droplets break or burst [24,33]. The key physical parameters for droplet deformation and interfacial tension are viscosity related properties such as $\eta_{c}$, $\eta_{d}$ and $\eta_{e}$, and strain rate $\dot{\varepsilon }$.

## 3. Experimental Methods

**Figure 2:**
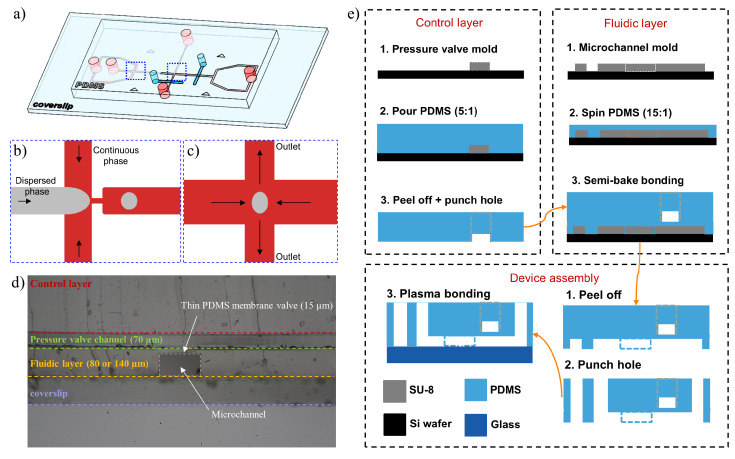
Microfluidic platform for droplet trapping at the cross junction: (**a**) 3D view of the microfluidic device, showing the slide glass, double-layered PDMS microchannels with the fluidic layer (in red) and the control layer (in cyan), (**b**) droplet generation at the flow-focusing region, (**c**) droplet trapping and stretching under extensional flow, (**d**) cross-section of the double-layered PDMS device and (**e**) step-by-step procedure of fabricating the double-layered PDMS device, highlighting features in a cross-section view.

We use standard multilayered soft-lithography protocols for fabricating the microfluidic device [37,38]. A more detailed fabrication procedure can be found in our previous work [38]. The microfluidic channel consists of a flow-focusing region to produce emulsion droplets and a cross junction region where the droplet is trapped and deformed under extensional flow, with precise on-chip pressure control (Figure 2a). The two pneumatic pressure valves are depicted in cyan color. The pressure regulation of the valves is achieved by using a pressure regulator (Proportion-Air, QPV1), with the maximum pressure of 150 psi and ± 0.1% accuracy of output pressure. The output of the pressure regulator is connected to the inlet port of the upper layer (control layer) in the device. The pressure regulator changes the flow resistance in one of the outlet channels, redistributing the planar extensional flow pattern, thus relocating the position of the stagnation point at the cross junction.

Emulsion droplets are first generated at the flow-focusing region by manipulating the flow rates of the continuous $\left(Q_{c}\right)$ and dispersed phases $\left(Q_{d}\right)$. The droplet size and distance between the droplets are controlled by the flow rate ratio $\left(Q_{c}/Q_{d}\right)$ (Figure 2b). The dispersed and continuous phases are 50 wt% aqueous glycerol and oleic acid, respectively. An excessive amount of 1 wt% span 80 was added into the continuous phase for generating stable droplets and providing an identical surfactant environment around the droplet. Through an active feedback control of the droplet position by using the pressure valve (depicted in cyan color in Figure 2a), droplets can be trapped and stretched under extensional flows with different strain rates at the center of the cross junction (Figure 2c). The thin PDMS membrane valve in a double-layered PDMS device is pressurized and bends down towards the microchannel by the air pressure, changing the resistance in one of the outlet channels (Figure 2d). Two fluidic layer thicknesses are used, channel I with *h* = 80 μm for 2D confinement of the droplet and channel II with *h* = 140 μm to eliminate geometrical confinement of the droplet under extensional flows. The two layers were aligned and assembled as shown in Figure 2e. Note that the droplet trapping can become difficult when the distance between droplets is too short due to drop-drop interaction.

## 4. Results and Discussion

### 4.1. 2D Darcy Approximation Model for Confined Droplet

**Figure 3:**
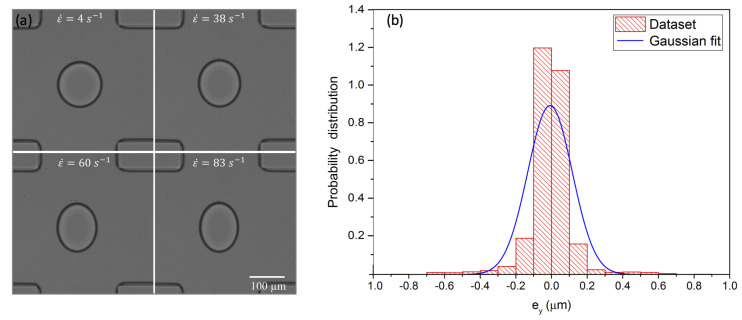
(**a**) Droplet deformation under different strain rates in a microchannel with channel height h=80μm, (**b**) probability distribution of the droplet position at strain rate $\dot{\varepsilon }$ = 83 s${}^{-1}$.

When the droplets of a dispersed phase are produced at the flow-focusing region, they travel downstream towards the cross junction. The droplets produced in the channel are confined into the channel walls as the confinement ratio δ becomes larger than unity. For the confined droplet in the 2D extensional flow, we use the shallow channel I with *h* = 80 μm in which $\delta =2R_{0}/h=2\left(65.6\pm 0.6\right)/80=1.64\pm 0.015$. Once the droplet is trapped in the cross junction of the channel by using the active pressure control system, only the continuous phase is introduced in the channel to avoid further droplet generation and drop–drop interaction. It was demonstrated in our previous studies that the steady hydrodynamic trapping of droplets was made possible by creating a potential well using the extensional flow and regulation of the pressure by the pressure control [38]. For the confined droplet case, the strain rate is increased from 4 to 83 s${}^{-1}$ to induce the droplet deformation once the droplet is trapped in the cross junction region of the channel (Figure 3a). At strain rate $\dot{\varepsilon }$ = 83 s${}^{-1}$, the right bottom image in Figure 3a displays the most deformed droplet in the cross junction.

The hydrodynamic trapping mostly relies on the extensional flow at the cross junction by applying appropriate pressure using the pneumatic valves to confine the droplet close to the stagnation point (zero-velocity and zero-velocity-potential), wherein the velocity potential $\phi =\left(\dot{\varepsilon }/2\right)\left(x^{2}-y^{2}\right)$ and the velocity $\vec{u}=\left(\left(\partial \phi /\partial x\right)\hat{i},\left(\partial \phi /\partial y\right)\hat{j}\right)=(-\dot{\varepsilon }x,\dot{\varepsilon }y$) [39,40]. Here, $\dot{\varepsilon }$ is the strain rate ($s^{-1}$), *x* and *y* are the spatial coordinates. For the droplet trapping with the active feedback control, we can transfer the minimum zero-velocity-potential point into the user-defined trapping position along y-direction. The center of the region of interest ($x_{t}$, $y_{t}$) corresponds to the user-defined trapping position of the droplet. The center of the droplet ($x_{c}$, $y_{c}$) is directly obtained by the image processing and considered as the current position of the droplet. The trapping error was defined as $e_{x}=x_{t}-x_{c}$ and $e_{y}=x_{t}-x_{c}$ along the x- and y- directions. For perfect trapping, the error distribution should represent a sharp peak at $e_{y}=0$. Our experimental setup shows that the droplet trapping was stable with the minimum trapping error as small as $e_{y}=\pm$ 0.5 μm at $\dot{\varepsilon }=83$ s${}^{-1}$ (Figure 3b). With the minimum trapping error, the droplet experiences symmetric extensional flow around it, which results in the homogeneous extension of the droplet.

**Figure 4:**
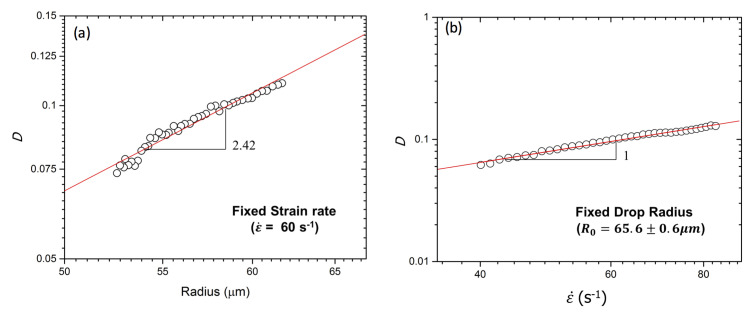
Droplet deformation *D* in a microchannel with channel height *h* = 80 μm: (**a**) Dependence of the droplet size at a constant strain rate $\dot{\varepsilon }$ = 60 s${}^{-1}$, (**b**) dependence of the strain rate at a constant droplet radius $R_{0}=65.6\pm 0.6$ μm. The symbols and the solid lines correspond to experimental data and power law fits, respectively.

The droplet deformation *D* is dependent on the viscosities of dispersed and continuous phases, interfacial tension, strain rate and droplet confinement. *D* is measured by varying the strain rate and droplet confinement at fixed viscosities and interfacial tension of fluids. A recent report has shown that droplet shape relaxation in the moderately confined regime exhibited strong dependence on the droplet radius but not on the ratio of dispersed to continuous phase viscosity [31]. We also observe that droplet deformation *D* increases with increasing droplet radius at fixed $\dot{\varepsilon }=60$ s${}^{-1}$ (Figure 4a). Similarly, *D* increases with increasing $\dot{\varepsilon }$ at fixed $R_{0}=65.6\pm 0.6$ μm (Figure 4b). It is reported [22,32] that *D* typically follows a linear trend on a log–log scale of $D∼R_{0}^{\alpha }{\dot{\varepsilon }}^{\beta }$. In our experiments, based on the best curve fit, α∼2.42 for different droplet radius at fixed $\dot{\varepsilon }=60$ s${}^{-1}$ and β∼1.0 for different strain rates at fixed $R_{0}=65.6\pm 0.6$ μm (see solid red lines in Figure 4a,b). From Taylor’s work for the droplet deformation upon shear flow [33], the droplet deformation is found to vary linearly with Ca when Ca<0.1. In our work, when $\dot{\varepsilon }$ is in the range of 4–83 s${}^{-1}$, Ca is on the order of $10^{-3}$ to $10^{-2}$ (with *v* = 0.009–0.018 m/s, $\eta_{c}$ = 0.037 Pa·s, σ=0.01–0.03 N/m), hence Taylor’s droplet deformation theory is valid. As expected, Taylor’s result is in good agreement with our results of $D∼{\dot{\varepsilon }}^{1.0}$. The dependence of *D* on the droplet size ($D∼R_{0}^{2.42}$) is also found to be similar to Ulloa’s work that considers the geometrical constraints on the droplet deformation [32].

**Figure 5:**
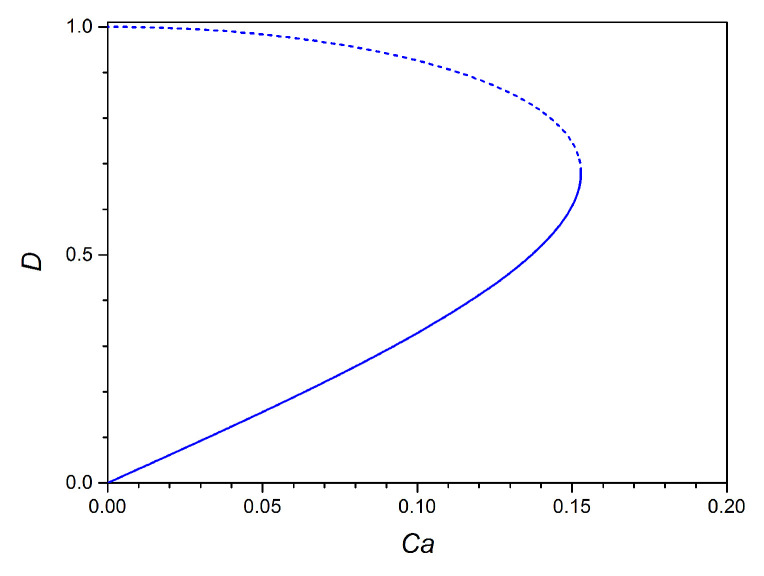
Numerical solution of Equation (4). The lower branch (solid line) corresponds to the physical solution, while the upper branch (dashed line) is unphysical.

We perform numerical predictions of the deformation of confined droplets by using the 2D Darcy approximation model at a fixed confinement ratio δ=1.64. By solving Equation (4), we obtain the droplet deformation *D* as a function of Ca (Figure 5). The unique solution of *D* is expected to show an upper branch (unphysical solution) and a lower branch (physical solution) with a critical *D* between them. The solution does not exist when D>0.68, which is related to bursting of the droplet [32,36]. At low Ca, the droplet deformation follows a linear trend. The linear trend at low Ca was also provided by other researchers for unconfined 3D spherical droplets numerically and experimentally [24,41]. However, the confinement ratio δ alters the slope of the linear relationship between *D* and Ca. It is found that the slope at low Ca in 2D model is slightly higher in comparison with the unconfined 3D droplet deformation [24,41], which results from the confinement effect that squeezes and deforms droplets more than the unconfined case.

### 4.2. IFT Measurements in Microfluidics with 2D and 3D Models

**Figure 6:**
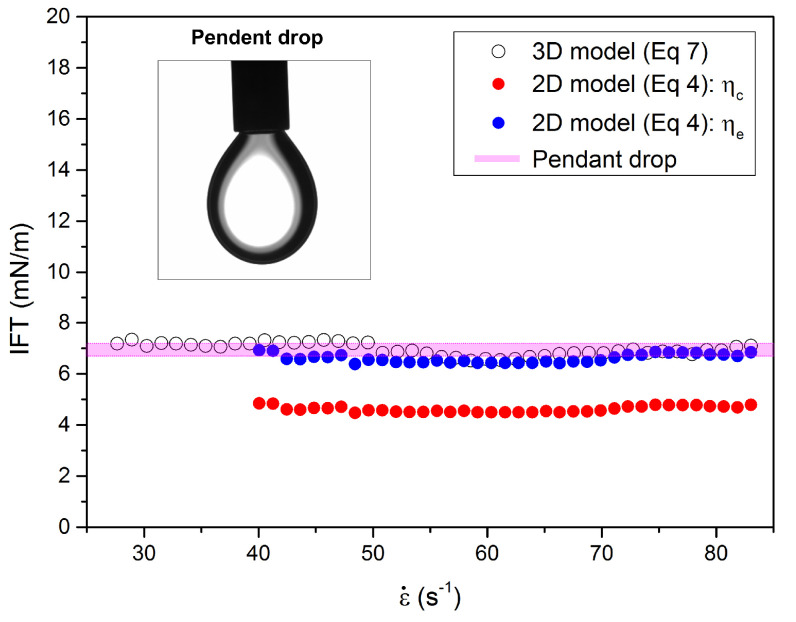
Interfacial tension results obtained by three different methods. A conventional pendant drop method is employed (colored in pink region). The interfacial tensions are compared with quasi-static 2D Darcy approximation (Equation (4)) and 3D small deformation models (Equation (7)).

The droplet deformation in a microfluidic platform allows one to measure IFTs of immiscible liquid pairs by considering the droplet deformation, confinement ratio, continuous and dispersed phase viscosities based on the force balance between extensional stress and liquid–liquid interfacial tension. We validated the quasi-static 2D model for the measurement of IFTs and compared the results with a conventional pendant drop method (Figure 6).

For the 2D Darcy approximation model, we calculated the IFT based on Equation (4). The key physical parameters affecting the IFT are $\eta_{e}$, $\eta_{c}$, $\dot{\varepsilon }$ and *D*. The strain rate $\dot{\varepsilon }$ was in the range of 40.1–83 s${}^{-1}$ by varying the velocities of continuous phase from 0.009–0.019 m/s. The deformation factor *D* measured under the different strain rates in Figure 4b was used to obtain Ca from Figure 5. The viscosities of $\eta_{e}$ and $\eta_{c}$ were measured by using a strain-controlled rheometer (ARES-G2, TA instruments) with a cone-plate geometry (50 mm in diameter and 1° truncation angle). $\eta_{e}$ and $\eta_{c}$ were used to calculate the interfacial tensions $\sigma_{1}=\eta_{e}\dot{\varepsilon }R_{0}/Ca$ and $\sigma_{2}=\eta_{c}\dot{\varepsilon }R_{0}/Ca$. When the effective viscosity $\eta_{e}$ is considered, $\sigma_{1}$ is calculated to be 6.6 ± 0.17 mN/m (blue symbols in Figure 6) that is similar to the one from the pendant drop method value of 6.95 ± 0.25 mN/m (pink symbols in Figure 6) with some minor discrepancy, that the droplet is confined in the microchannel walls with the confinement ratio of δ = 1.64 ± 0.015 while the theoretical model assumes an infinite 2D droplet deformation along z-axis. When the continuous phase viscosity is considered in Ca, $\sigma_{2}$ is much smaller than the value obtained by the pendant drop method (see red symbols in Figure 6). Our results indicate that the quasi-static 2D Darcy approximation model is a useful tool for real-time and in-situ interfacial tension measurements for certain applications such as membrane emulsification [42] and terrace-based microchannel emulsification [43] processes where the channel geometry is fixed, and the droplet sizes are comparably larger than the channel height.

For the 3D small deformation model, the IFT of the droplet is measured by using Equation (7). The IFTs are measured by varying the velocities of continuous phases from 0.0045–0.019 m/s that correspond to the strain rates from 25 to 83 s${}^{-1}$. The values of IFTs are calculated to be 6.97 ± 0.23 mN/m (black open symbols in Figure 6) that agree well with the one measured by the pendant drop method. There are some fluctuations of the IFT values while changing the strain rate, which may result from experimental error during image analysis of the droplet since IFT can vary up to 15% when the deviation of the major or minor radii of the droplet reaches 1% [44].

To summarize, we employed the microfluidic approach with confined and unconfined droplet deformations under the quasi-static 2D and 3D extensional flows to measure interfacial tensions of two immiscible fluids. The droplets undergo deformation upon the extensional flows. The 2D Darcy approximation model with the effective viscosity gives a reasonable IFT value even though the model cannot describe the complex 3D effects that occur at the interface between immiscible fluids. The 3D small deformation model also showed good estimation of IFT values when compared to the conventional pendant drop result. These models are based on the force balance between the interfacial tension force and the drag force acting on the droplet. It is advantageous that the influence of confinement extends the utility of this approach to IFT measurements since a single device can be used to study a wide range of droplet sizes. Practically, droplets confined in shallow geometries can be simplified as a disc shape in which the confinement effect plays a key role in the deformation and dynamics of droplets. The 2D deformation model is also useful to understand the confinement effect since the presence of the confinement alters the droplet deformation. However, the 2D model is not desirable when there is a strong interaction between the confining walls and droplets or strong charges exist on the walls. On the other hand, the 3D model requires that the microfluidic channel dimensions are larger than the droplet size. If the droplet size is close to the channel height, it can lead to incorrect prediction of low IFT [20]. In addition, more robust calculation in the 3D model allows one to give a more accurate prediction for IFT. Nevertheless, both the 2D and 3D models by microfluidic approaches are very useful to investigate the dynamics of droplets such as surfactant adsorption onto droplets, droplet aging, transient interfacial dynamics owing to increased residence times at the cross junction under quasi-static extensional flows. Especially, our microfluidic tensiometry can capture transient interfacial tension at milliseconds with small sample volume, minimum quantities of chemical reagent and sample waste, as well as high accuracy of interfacial tension measurement and interfacial reaction occurring on the interface. Our platform also allows one to capture phase change and nanoscale phase separation which take place by controlling the kinetics and thermodynamics of film formation and/or molecular chain structure. We expect our platform to become valuable for studying multiphase flows and dynamics, which are ubiquitous in many industrial and biochemical processes and applications, such as filtration and precipitation, food, cosmetic and pharmaceutical industries [6,7,12,13].

## 5. Conclusions

This work investigated the droplet deformation in both quasi-static 2D and 3D extensional flows in a microfluidic platform and measured the interfacial tensions of immiscible liquids based on droplet deformations. The droplets are trapped at the stagnation point with the aid of the active feedback control of the pressure valves, and stretched along the outflow direction upon the extensional flow. IFT calculation based on the 2D Darcy approximation with the effective viscosity shows good agreement with the pendant drop method. This 2D droplet deformation model is a useful tool to measure interfacial tensions in-situ and in real time in certain applications where the channel geometry is fixed and the droplet sizes are comparably larger than the channel height. The 3D small deformation model captures the interfacial force balance on the droplet surface, which gives more robust theoretical calculation than the 2D model. We confirmed that both 2D and 3D models provide good interfacial tension measurements in comparison with the conventional pendant drop method. This work will help develop not only the generation of emulsions on chip but also incorporate knowledge to conduct in-situ, real time interfacial tension measurement on a single platform. Further, it will provide valuable insights to understand the confined and unconfined models for the droplet deformation and interfacial tension dynamics of immiscible liquids in droplet microfluidics.

## Figures and Tables

**Table 1:** Mathematical symbols and their definitions used in this study.

Symbol	Definition
*A*	Area of the droplet
*b*	Smallest distance of the droplet surface from its center
Ca	Capillary number
*D*	Droplet deformation
δ	Channel confinement parameter
$\dot{\varepsilon }$	Strain rate
$\eta_{c}$	Continuous phase viscosity
$\eta_{d}$	Dispersed phase viscosity
$\eta_{e}$	Effective viscosity
$\hat{\eta }$	Relative viscosity
Γ	Droplet interface
*h*	Channel height
*l*	Largest distance of the droplet surface from its center
*p*	Pressure
ϕ	Velocity potential
ψ	Stream function
$Q_{c}$	Flow rate of the continuous phase
$Q_{d}$	Flow rate of the dispersed phase
$R_{0}$	Initial droplet radius
σ	Interfacial tension
*t*	Time
*u*	Droplet velocity
$\vec{u}$	Depth-averaged velocity of fluid around the droplet
*w*	Complex potential
